# A bibliometric study on the research outcome of Brazil, Russia, India, China, and South Africa

**DOI:** 10.12688/f1000research.51337.1

**Published:** 2021-03-16

**Authors:** Aparna I Narayan, Bharti Chogtu, Manthan Janodia, Santhosh Krishnan Venkata

**Affiliations:** 1 UniARC Services LLC, Udupi, Karnataka, India; 2 Manipal College of Dental Sciences, Manipal Academy of Higher Education, Manipal, India; 3 Kasturba Medical College, Manipal Academy of Higher Education, Manipal, India; 4 College of Pharmaceutical Science, Manipal Academy of Higher Education, Manipal, India; 5 Manipal Institute of Technology, Manipal Academy of Higher Education, Manipal, India

**Keywords:** BRICS, Collaboration, Scopus, Field Weighted Citation Impact

## Abstract

**Background**: Publication is one of the quantitative measures of countries' contribution to research and innovation. This paper attempts to understand the publication related information of the BRICS countries (Brazil, Russia, India, China, and South Africa).

**Methods**: Detailed analysis of publications on the basis of collaboration, research area, number of publications, percentage of Gross Domestic Product (GDP) spent on research, and citation is presented in the paper. An attempt is also made to understand the relations between each of the parameters and the overall performance of the country.

**Results**: Times Higher Education global ranking is considered as a measure to validate the claims of this paper.

This study shows that among the BRICS nations, China with the highest percentage of GDP spent on research has also the highest number of researchers and publication output whereas South Africa excels in terms of number of international collaborative publications and publications in high impact journals. This article has highlighted the distribution of publications as per the subject area with India leading in the area of Computer Science.

**Discussion**: Results showed a strong relationship between each of the parameters discussed on the research performance of a country.

## Introduction

The BRICS (Brazil, Russia, India, China, and South Africa) countries are emerging as major influencers of geopolitical changes. BRICS was referred to for the first time in 2001 in a report titled Building Better Global Economic BRICS by Goldman Sachs economist Jim O’Neill. With the success of BRICS over the years, scientific literature has tried to have an improved understanding of these economies. ^1^ In 2011 a total of 17.3% of research papers in science and engineering were published by BRICS countries compared to just 7.6% in 1995. This accounted for a 233.4% increase in publication output in 2011 compared to 1995. ^2^ Globally, Brazil ranked 13th in terms of peer-reviewed papers produced between 2011 and 2016. Expenditure on research and development (R&D) as a percentage of Gross Domestic Product (GDP) was 1.17% in 2014 whereas citation impact has remained below average during the same period but improved between 2011 (0.73) and 2016 (0.86), an increase of 18%. ^3^ Russia had set aside a budget of 70 billion roubles (approx. US $3 billion) in 2018 while doubling its scientific paper output between 2006 and 2016. ^4^ A total of 74,697 papers (research, review articles, and conference papers) were published by the Russian Federation in Scopus-indexed journals with a compound annual growth rate (CAGR) of 8% between 2006 and 2016, more significantly between 2012 and 2016 with a CAGR of 15.2%, while 54,669 papers were published in Web of Science (WoS) journals with CAGR of 5.9% during 2006-2016 and 12.9% between 2012-2016. ^5^ Russia and China are predominantly single funding agencies, whereas Brazil and India have diversified funding sources. It was also found that research publications from Russia with foreign funding received more visibility and are published in journals with an average mean impact factor 1.9 times greater than Russian-funded publications in WoS. ^6^ Russia spends 1% of national income on R&D, whereas public institutions conduct about 75% of research. ^7^ In recent years, China is second only to the United States (US) in terms of R&D investment. It spent around 2.08% of the national GDP on R&D in 2015. Chinese Academy of Sciences (CAS), Ministry of Science and Technology (MOST), and National Natural Science Foundation of China (NSFC) are the three major funding agencies in the country. ^8^ Based on recent data, South Africa spent about 0.8% of GDP on R&D.

The government of South Africa also aims to increase this spending to 1.5% of GDP in the ensuing decade. ^9^ In the five-year block of 2013-2017, researchers from South Africa increased their average growth rate in WoS publications by 13.12%, almost double that of the world average of 6.36%. ^10^ South Africa as a country is ranked 18th in the world in social sciences and arts and humanities; 33rd in life sciences; 38th in physical sciences and 40th in technology out of 198 countries studied. However, the total number of papers published by South Africa during 1995-2016 is approximately 31,651 for social sciences and 37,847 for technology. Though by sheer number the technology domain has produced more papers, the ranking for this domain for SA is way below 18 for social sciences with a much lesser number of papers. Similarly, for the arts and humanities field, with 8,842 papers South Africa’s global rank was 18. ^11^ The journal publication output in 2016 increased by 4.5% compared to 2015, which was still lower than 6.4% during 2014-15. The majority of papers from researchers were in the fields of science, engineering, and technology, which accounted for 58.44% of total publications. ^12^ R&D expenditure for India tripled in the last decade; from Rs 24,117 crores in 2004-05 to Rs 85,326 crores in 2014-15. However, as a percentage of GDP, the R&D expenditure remained stagnant at 0.7% during the same period. Of total investment in R&D, the public sector has contributed between 0.4-0.5%, whereas 0.2-0.3% came from the private sector. Strikingly for India, the government is also a user of a research fund; apart from playing the role of the largest funder, the major chunk of R&D expenditure is used by the Central government. In addition, to increase nominal R&D expenditure, India’s share of total publication output also increased during 2009-2014 with a growth of 14% during the said period; an increase in the share of total global publications rose from 3.1% in 2009 to 4.4% in 2014. India was also ranked 6th in terms of total scientific publication output globally with a growth rate of 13.9% (Scopus) and 7.1% (Science Citation Index - SCI) compared to the world growth rate of 4.4% and 4.1%, respectively. ^13, 14^ The number of researchers per million population increased from 110 in 2000 to 218 in 2015. ^14^

BRICS represents about 42% of the world’s total population. 31.2% of science and engineering doctorates globally are produced by BRICS. ^15^ BRICS countries together accounted for an impressive 233.4% growth in 2011 over 1995 in publishing science and engineering research papers, whereas the growth rate for other countries was 31.3%. ^16^ Other than Russia, the output of BRICS countries increased for most frequently cited papers at a higher rate than top-cited countries globally. ^17^ The co-authored papers among the BRICS countries are found to be countries with which they have “geographical” “cultural” and/or “historical” proximity. ^18^ The intensity of intra-BRICS countries’ collaboration is also low. ^19^ Further, the strength of each BRICS country is in the different scientific domain in terms of their research output according to the WoS database for the period 2008-2011. Research output from Brazil was primarily in agricultural, plant and animal sciences; for Russia it was physics, chemistry, mathematics and geosciences; India’s research outputs were in the fields of pharmacology and toxicology, agricultural sciences, material sciences, and chemistry; while China’s publications were in the fields of material sciences, chemistry, and physics. South Africa’s research output focused more on the fields of plant and animal sciences, immunology, environment/ecology as well as multidisciplinary research areas. Quantitatively, China is the largest in terms of publication output, whereas South Africa is the smallest among the five BRICS countries. Alternatively, based on Scopus data for the year 2015, China took the lead in the subject areas of mathematics, physics and astronomy, multidisciplinary science, and earth and planetary science. For Brazil, the strongest subject area according to Scopus is dentistry; for Russia, it is physics and astronomy; India is strong in the fields of pharmacology, toxicology, and pharmaceutics; and for South Africa, the strongest subject area is agricultural and biological sciences. ^19^ Based on Scopus data, the total publication output of BRICS countries in 2015 was 29% with China contributing 18%, India 5%, Russia and Brazil 2.6%, and South Africa 0.72%. ^19^ From the above discussion, it is clear that existing literature has discussed the need for analysis of research outcomes of a country. From the literature study, it can be seen that BRICS nations are the emerging economies where research publications are gaining over the years. With a motive to understand the research scenarios of BRICS nations this study compares number of researchers and the research outcomes of these nations in terms of research spent, citations, collaborative publications, distribution of publications across different subject areas and percentage of high impact publications.

### Background

**Figure 1.**
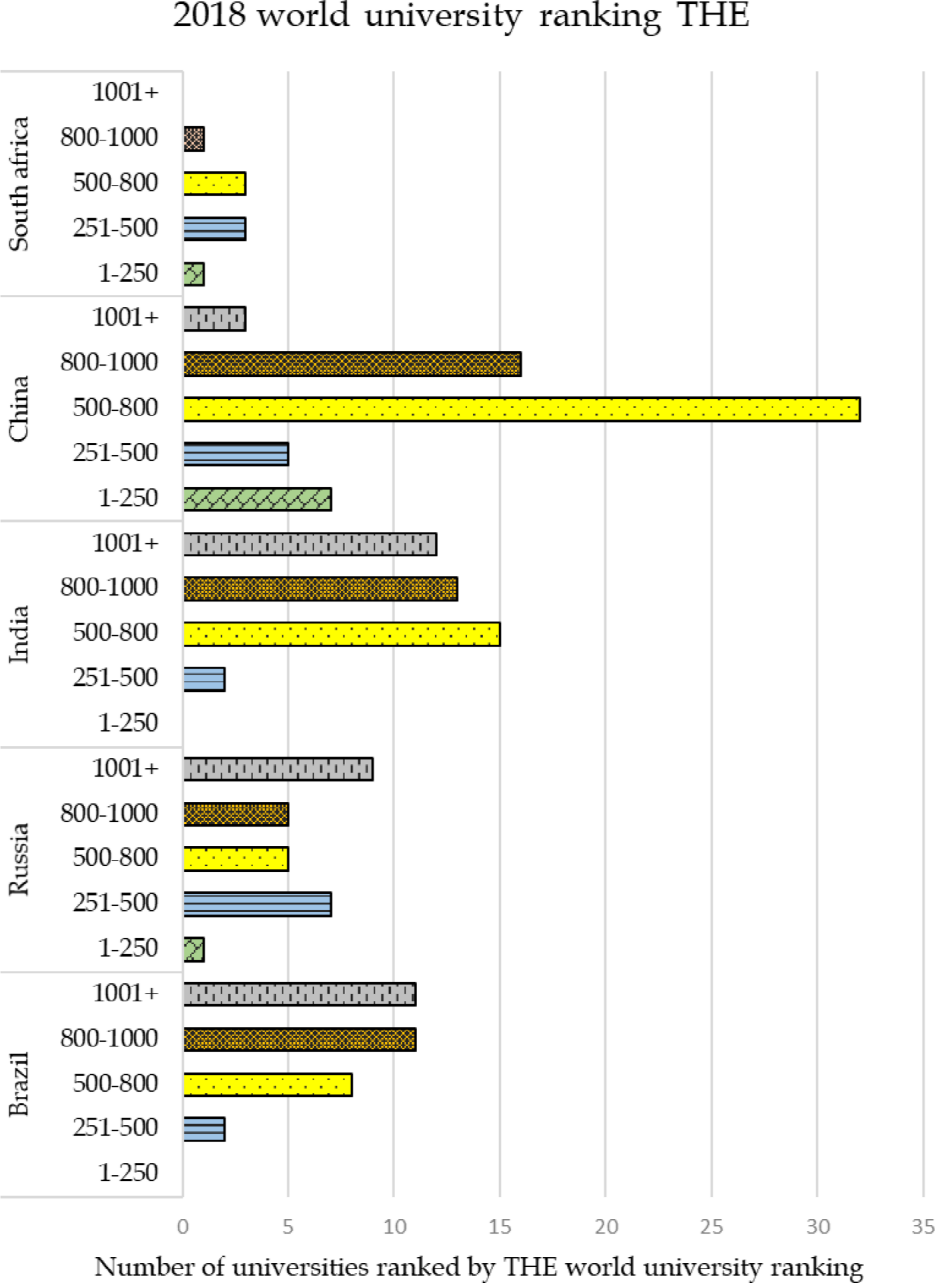
Distribution of ranking country wise in THE world university ranking 2018.

**Figure 2.**
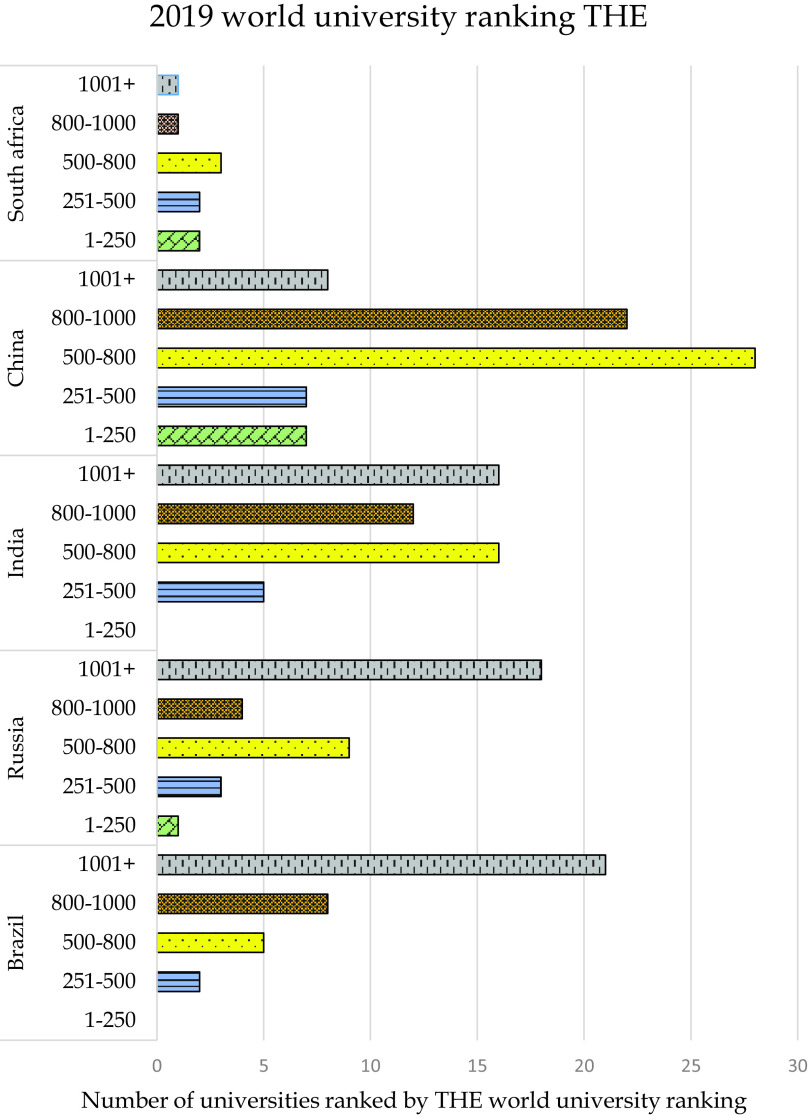
Distribution of ranking country wise in THE world university ranking 2019.

The ranking of universities has always been a topic of debate, but it is a necessary mechanism to understand the performance of the universities across the globe. There are multiple ranking agencies that rate higher education institutions across the globe such as the QS world ranking, Times Higher Education (THE) world university ranking, Shanghai university ranking, Leiden ranking, etc. THE is one of the widely accepted ranking agencies that ranks the higher education institutions based on four basic parameters, which are teaching, research, international outlook, and industry interaction, out of which research contributes almost 62.5% of the overall scores, thus making it important for the proposed work to analyze the outcomes concerning research. Considering the emerging economies (BRICS), classification is made across the ranking to project the distribution of the number of universities across the entire scale. For this study we have classified the ranking window into five different ranges, which are 1 to 250, 251 to 500, 500 to 800, 801 to 1000, and more than 1000. Figure 1 shows the distribution of universities of BRICS nation across these five ranges for the 2018 ranking and Figure 2 shows the distribution for 2019 ranking. It can be seen that from Brazil, 32 universities have been featured in the 2018 ranking and the number has increased to 36 for the year 2019 ranking. From Russia 27 universities feature in the year 2018, and the number increased to 35 for the 2019 ranking. India had 42 universities ranked in 2018 and the numbers rose to 49 for the year 2019 ranking. There were 63 Chinese universities ranked in the year 2018 and 72 universities ranked for 2019 and finally, from South Africa, eight universities feature in 2018 ranking and nine universities for the year 2019. From the trends, it can be understood that in all the emerging countries the number of universities ranked has increased by a minimum of 10% percent or more as compared to its previous years. From the figures, it can be seen that for the year 2018, universities from South Africa, China, and Russia were featured in the bracket of 1-250 with China leading the group by seven universities, with no universities from India and Brazil in that bracket. In the bracket of 251-500, all the nations have representation, with Russia as the leading country with seven universities followed by China with five universities, then by South Africa with three universities and both Brazil and India with two universities each in this range. The 501-800 bracket is the range in which the maximum number of universities are placed; China leads the list with 32 universities, followed by India with 15 universities, Brazil with eight universities, Russia with five universities, and South Africa with three universities. In the next bracket of 801-1000, China leads by 16 universities followed by India with 13 universities, Brazil with 11 universities, Russia with five universities, and South Africa with one university. In the last bracket of universities ranked more than 1001+ India leads by 12 universities followed by Brazil with 11 universities, Russia with nine universities, China with three universities, and no university from South Africa is ranked in this bracket.

The 2019 ranking also showed a similar trend with some slight modification in the behavior, which is due to an increase in the number of universities. Again, in the bracket of 1-250 only universities from China, Russia, and South Africa are featured with seven, two, and one universities, respectively. The 251-500 bracket has Chinese universities in the lead with seven universities, followed by India with five universities, Russia with three universities, Brazil and South Africa with two universities each. Similarly to 2018, the year 2019 also shows a big number of universities in the 501-800 range with China having 28 universities, followed by India with 16 universities, Russia with nine universities, Brazil with five universities, and South Africa with three universities. The next bracket of 801-1000 also had an increase in number as compared to the previous year, with China having 22 universities, followed by India with 12 universities, Brazil with eight universities, Russia with four universities, and South Africa with one university. The last bracket of 1001+ ranking shows Brazil leading the list with 21 universities, followed by Russia with 18, India with eight universities, and China with eight universities, and lastly South Africa with one. From the comparison of the two years, it can be seen that India and China have improved in numbers and also quality with not much change from Brazil, Russia, and South Africa.

To further understand the ranking of universities we attempt to analyze the research outcomes of the countries, as research constitutes 62.5% weightage in the computation of scores for ranking the universities. Several parameters contribute to research outcomes; a detailed analysis is presented in the following section considering parameters such as the number of researchers, percentage of GDP spend on research, number of publications, citations, collaboration, and area of research.

## Methods

To further analyze the reasons for the same we have first looked at the comparison of researchers’ population across the countries (Source: https://data.worldbank.org/indicator/SP.POP.SCIE.RD.P6?view=chart), Research and Development spend by % GDP (Source: https://data.worldbank.org/indicator/GB.XPD.RSDV.GD.ZS), and publication data from Scopus/ SciVal. Data related to researcher population for the years 2013 to 2017 is extracted from the world bank data, for computation the average value over the year is taken. Similarly for computation of research spend by percentage GDP is also extracted from the world bank data for the years 2013 to 2017 by taking the average over the period, all the data were extracted from the source site on 18 ^th^ December 2019. Publication related data corresponding to country output is extracted from the Scopus website on 18 ^th^ December 2019. Parameters related to the citation outcome, subject wise breakup and international coauthored publication details are analyzed from the extracted data. SciVal tool from the Elsevier is used to obtained score related to citation impact, international collaboration as obtained on 18 ^th^ Dec 2019. We also analyzed research output on three important parameters, namely researcher per million populations, total scholarly output, and expenditure on R&D as a percentage of GDP. The data on these three parameters were obtained from World Bank indicators. We compared these three parameters against the Field Weighted Citation Impact (FWCI) and its impact on collaborative publications with international authors. Based on three basic parameters, subject-wise analysis for BRICS countries’ research output was carried out.

## Results

**Figure 3.**
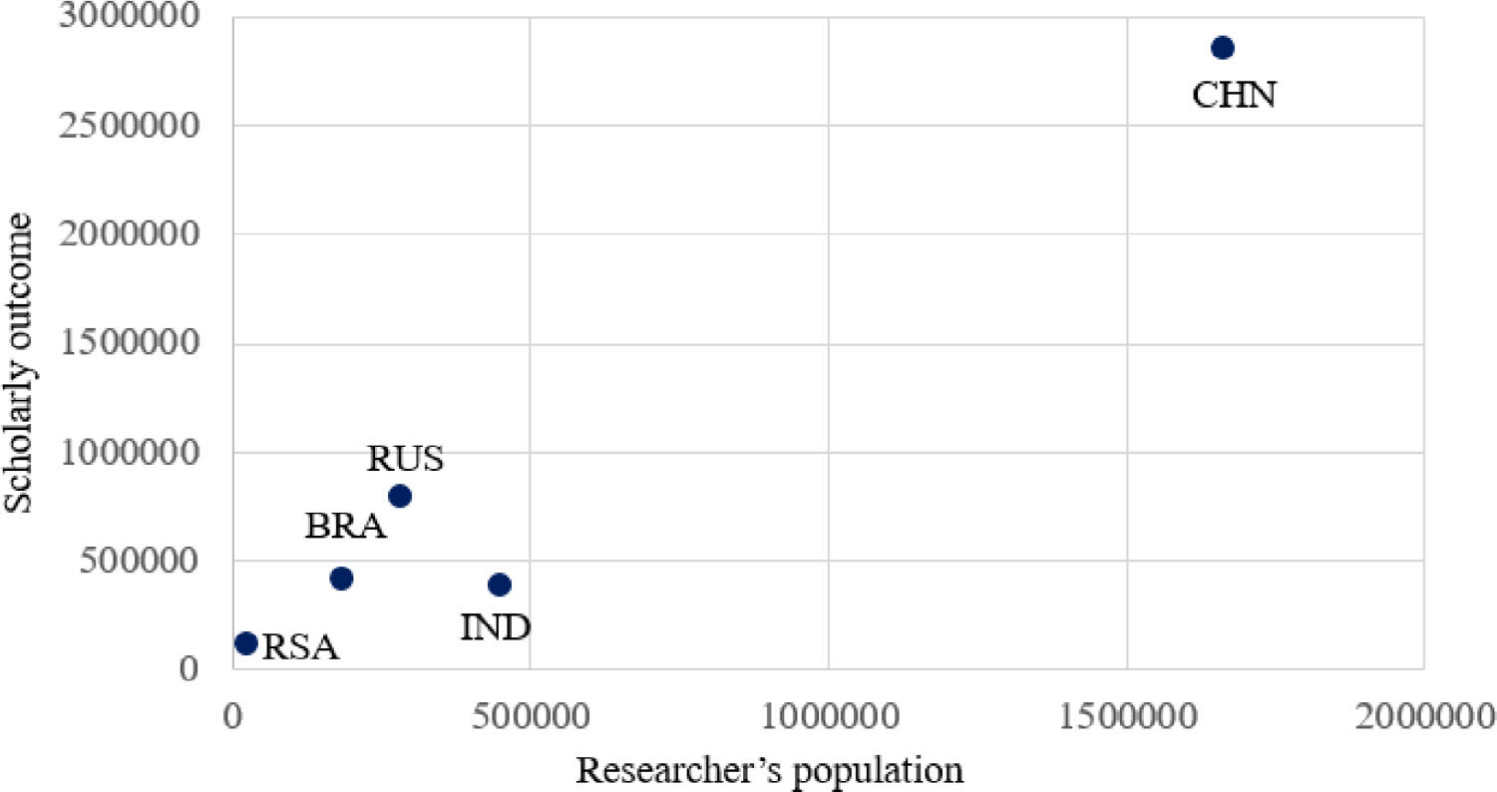
Comparison of scholarly outcome of countries with its researcher population. RSA: South Africa; BRA: Brazil; RUS: Russia; IND: India; CHN: China.

The researcher population is one of the important factors which determines the research strength of the institution or country. To understand the outcome in terms of publication in Scopus indexed publications for five years (2013-17) for the researcher population across the BRICS nations, a graph is plotted in Figure 3. From the plot, it is clear that China (CHN) with the largest researcher population of 1.67 million produces the highest quantum of publication of the 2.8 million. India (IND) has a researcher population of 0.43 million, producing around 0.48 million publications. Russia (RUS) produces around 0.8 million publications with around 0.35 million researcher population. Brazil (BRA) with a researcher population of 0.18 million produces around 0.48 million publications and South Africa (RSA) with a researcher population of 0.026 million produces around 0.05 million publications.

**Figure 4.**
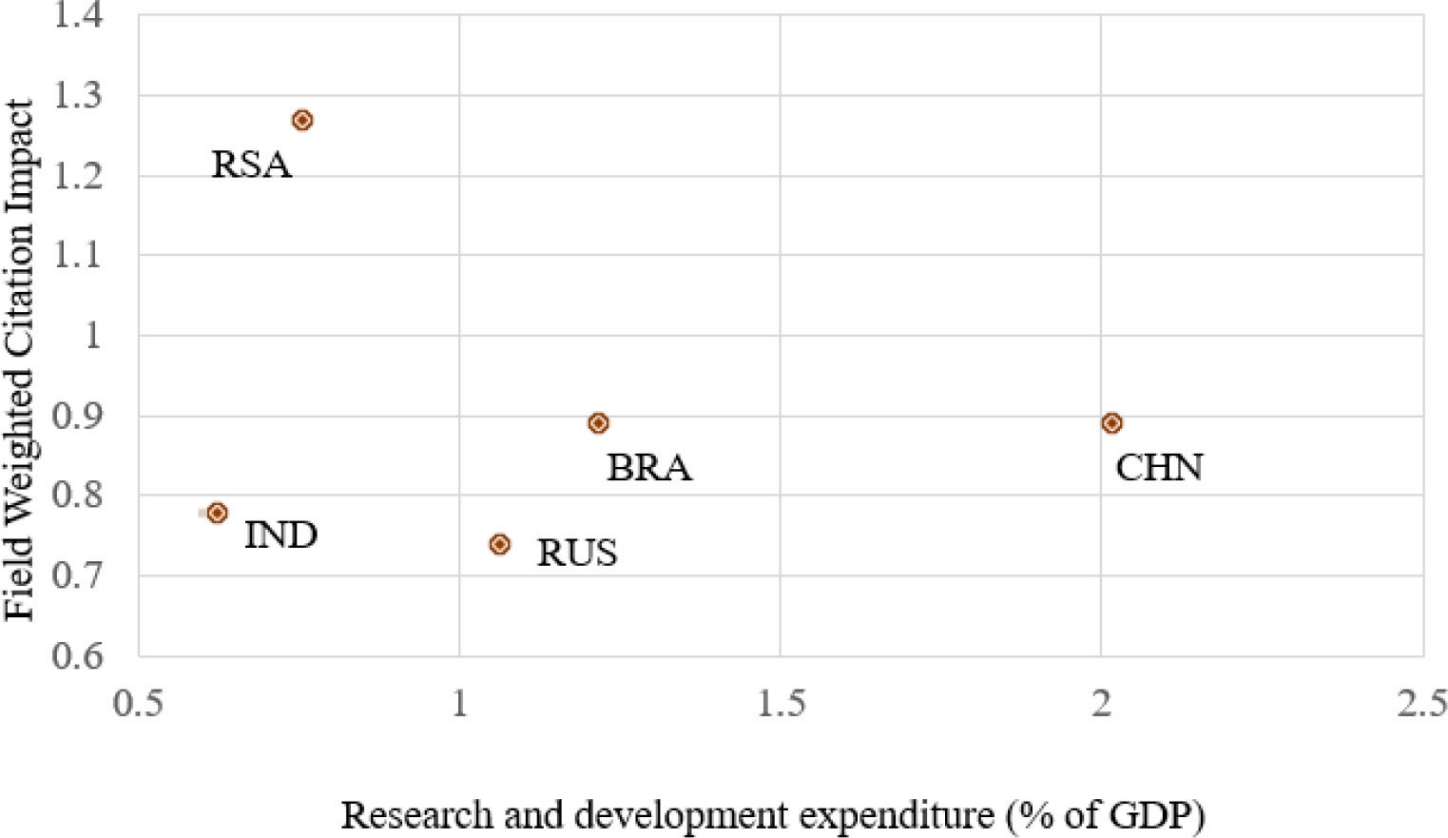
Comparison of publication quality with research and development expenditure. RSA: South Africa; BRA: Brazil; RUS: Russia; IND: India; CHN: China.

One more important factor which constitutes the growth of research is the percentage of GDP spent on research. Figure 4 shows the comparison of the percentage of GDP spent on research by countries to the citation index of the country (here we consider the FWCI as a citation metric as it is the normalized value of citation across the various subject areas). China has around 2.1% of its GDP spent on research, followed by Brazil with 1.27%, Russia with 1.07%, South Africa with 0.77%, and India with 0.6%, with a country average FWCI of 0.89, 0.89, 0.72, 1.27, and 0.78, respectively. From the figure, it is clear that the quality of research and the GDP spent has a direct relation except for the scenarios in India.

**Figure 5.**
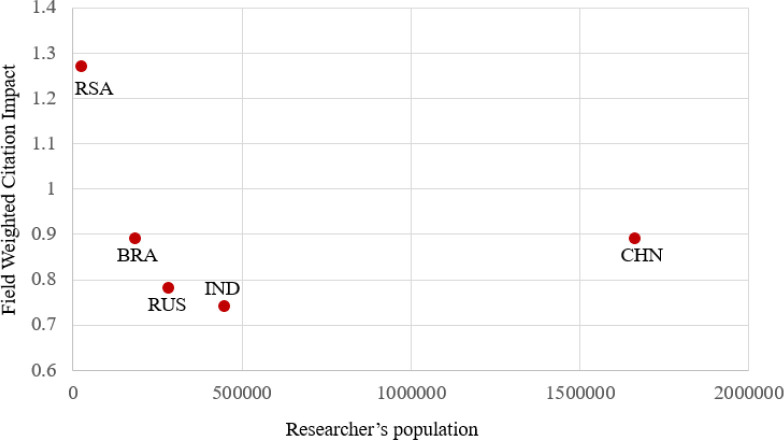
Comparison of publication quality with researcher population in the country. RSA: South Africa; BRA: Brazil; RUS: Russia; IND: India; CHN: China.

To have a picture of the quantity and quality of research, a graph was plotted in Figure 5 comparing the population of the researchers to the country average FWCI. It can be seen that China produces the highest number of publications, whereas South Africa with the least number of publications has the best quality citation of 1.27. Brazil, India, and Russia’s research outcomes and quantity of publications lie in this range.

From the above discussion, it can be seen that the quantity of research output, quality of research, researcher population, and research investment of the country are interrelated. To have a deeper understanding of the rest of the variables, the next section focuses on other parameters like the collaboration and subject area of the research.

### Organization of scholarly output

Data for publications that are indexed in Scopus for the period of 2012-17 are analyzed for the BRICS nations with different research metrics like the number of publications published year on year during the period of evaluation, followed by the impact of collaborative publication. Publication behavior across the different subject areas is studied and discussed in detail in this section.

**Figure 6.**
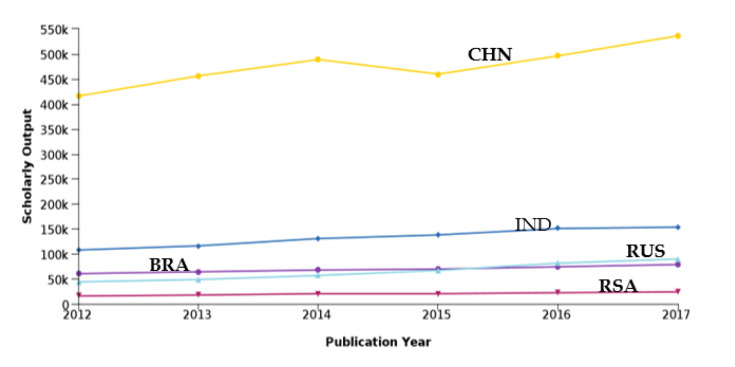
Countries Scholarly outcome year on year. RSA: South Africa; BRA: Brazil; RUS: Russia; IND: India; CHN: China.

Yearly scholarly output in Scopus-indexed publications of BRICS countries is plotted in Figure 6. It is seen that China produces the highest number of publications, which is much higher than the summation of the output of all other BRICS nations. India produces the next highest number of publications, which is almost 20% in comparison to China. Brazil and Russia produce almost the same number of publications. It can be observed that Brazil produced higher numbers of publications in comparison to Russia prior to 2015, but post 2015 the publication output of Russia is higher as compared to Brazil. South Africa produces the least publications as compared to all the other countries, a trend which is almost consistent over the period studied (2012-17). The quantity of publications produced by India constantly increases over the period 2012-17. As expected for the time period in 2015, the publication output of China was mostly moving in a positive direction. This increase in number may be relative to the increased number of researchers and also the higher research investment.

### Collaboration

**Figure 7.**
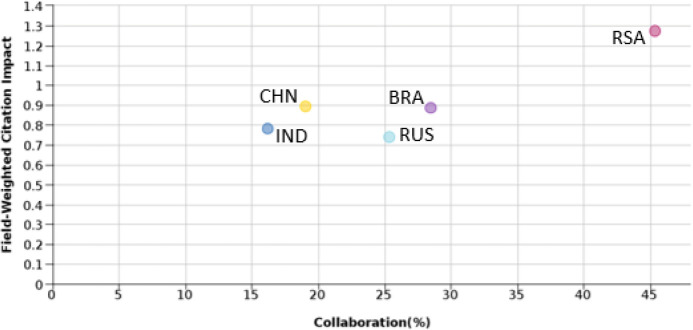
Comparison of publication quality with international collaborative publication. RSA: South Africa; BRA: Brazil; RUS: Russia; IND: India; CHN: China.

Collaboration in the publication would reflect the multicenter activity carried out by researchers. It is expected that research executed with multiple partners would have a relatively higher outreach and would lead to higher readership and citation. To understand this, Figure 7 shows the FWCI of BRICS nations in comparison to the percentage of publications with international collaboration for the time period 2012-17. From the figures, it is seen that South Africa has the highest percentage of international collaborative publication (as high as 45%) and also has the highest FWCI as compared to all BRICS nations. On the other hand, India has the lowest percentage of international collaborative publications and FWCI. China, Brazil, and Russia have a similar percentage of papers with international collaboration: 19%, 25%, and 28%, respectively. The FWCI of these nations is 0.9, 0.2, and 0.89, respectively. From Figure 7 it can be observed that India, Brazil, and South Africa have a similar trend. The behavior of Chinese and Russian characteristics is a little deviated, which may be due to other factors.

**Figure 8.**
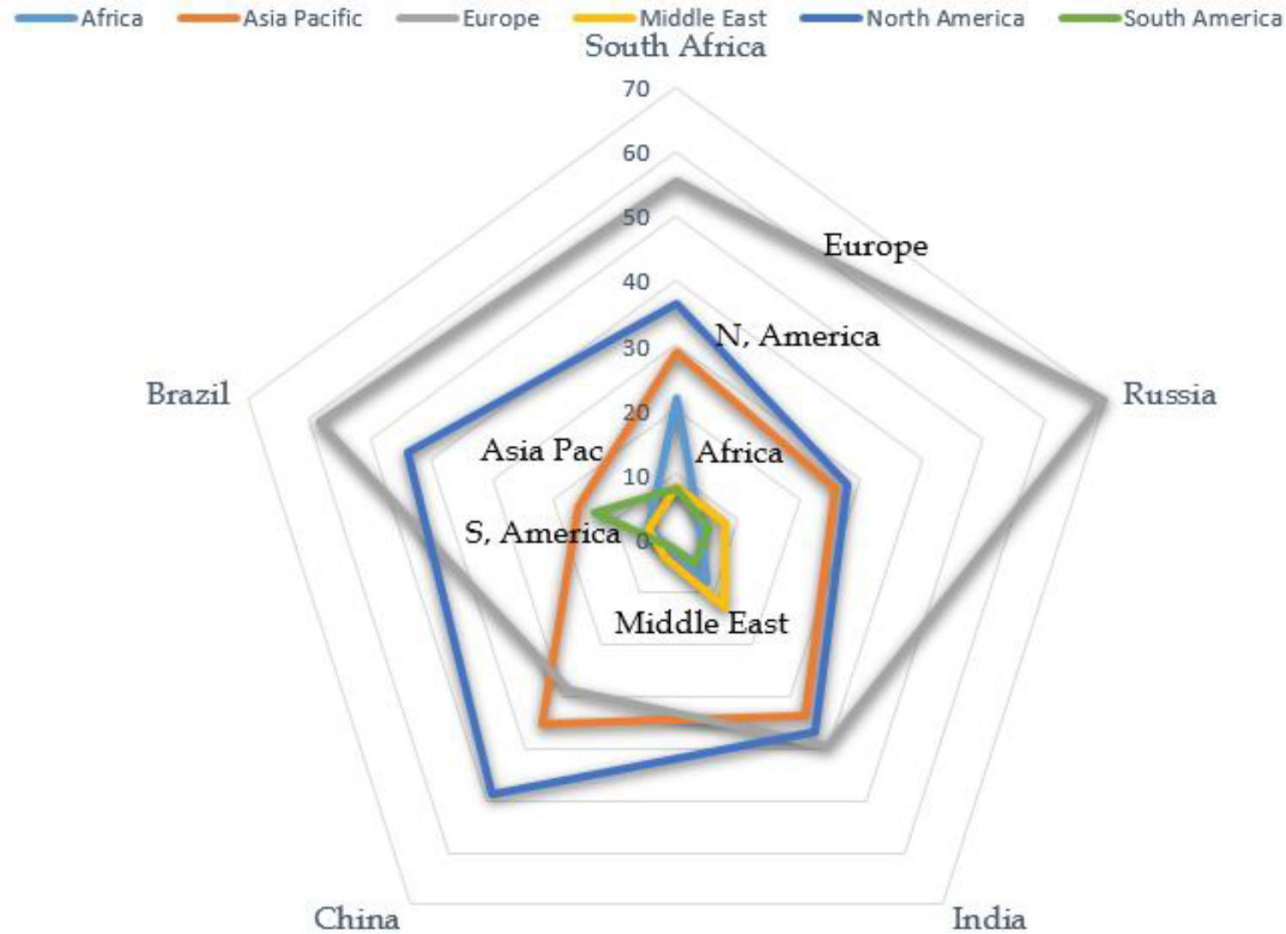
Distribution of collaborative publication of BRICS countries across regions.

An attempt is made to understand the distribution of the international collaborative papers published by BRICS among different regions across the globe as shown in Figure 8. For the analysis, the world is divided into six regions, namely Africa, Asia Pacific, Europe, Middle East, North America, and South America. Figure 8 shows the distribution of BRICS collaborative publications among these regions. It is observed that the highest percentage of collaborative publications for Brazil, India, South Africa, and Russia happens from the European region with 58%, 40%, 70%, and 58% of international collaborative publications, respectively. China has the highest percentage of collaborative publications from North America (50%). For the rest of the countries the North American collaborative publication share among collaborative papers is second; Brazil with 43%, South Africa with 38%, Russia with 29%, and India with 38%. With China again a different characteristic is observed; the Asia Pacific collaborative publication share is second with a share of approximately 36%. Based on percentage share of collaborative publications, the Asia Pacific region is in third position for Brazil, South Africa, Russia, and India with 8%, 30%, 26%, and 35%, respectively. European collaborative publication share is seen to be the third largest share of publications for China with 20%. For Brazil, the rest of the publications are shared with South America, the Middle East and the African region, with a percentage share of 15%, 1%, and 0.8%, respectively. In the case of South Africa, the rest of the collaborative publications are from the African region with 23%, followed by South America and the Middle East with almost 8% each. Russia's collaborative publications are shared 9% with the Middle East, followed by 8% with South America and 7.2% with Africa. India's collaborative publications with countries in the Middle East is around 16%, followed by 9% with African countries, and 7.5% with the South American region. Lastly, China has around 2% of papers in collaboration with the Middle East, South America, and Africa. Except for Russia where a large skew is present for European collaborative publication (70%), for the rest of the countries it can be seen that they have a similar sharing percentage.

### Relation of BRICS nations

**Figure 9.**
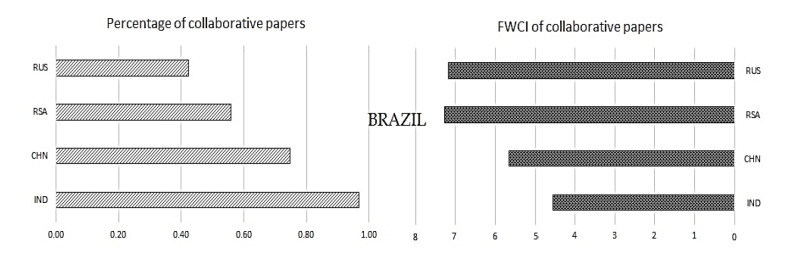
The interrelation of Brazil publication metrics with other BRICS countries.

**Figure 10.**
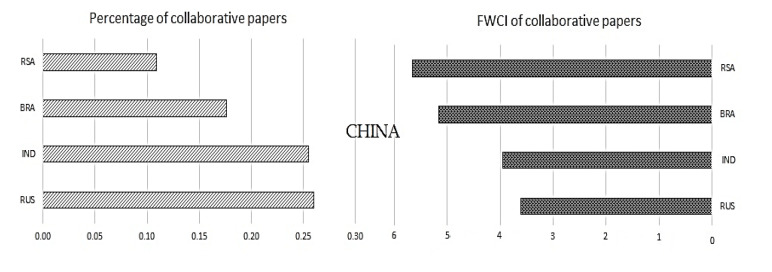
The interrelation of China publication metrics with other BRICS countries.

**Figure 11.**
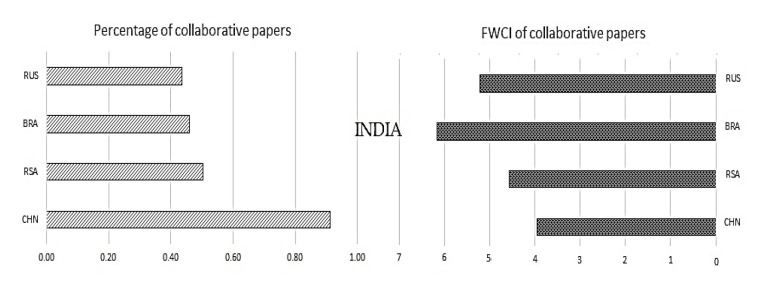
The interrelation of India publication metrics with other BRICS countries.

**Figure 12.**
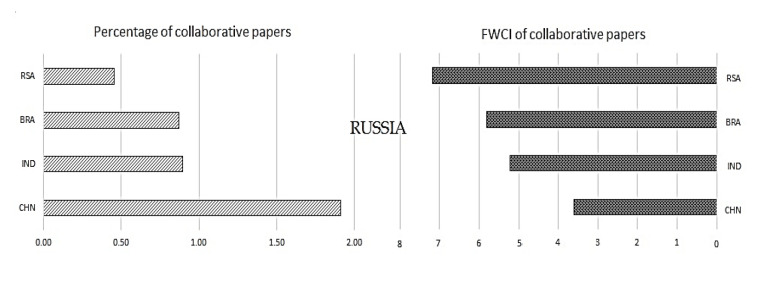
The interrelation of Russia publication metrics with other BRICS countries.

**Figure 13.**
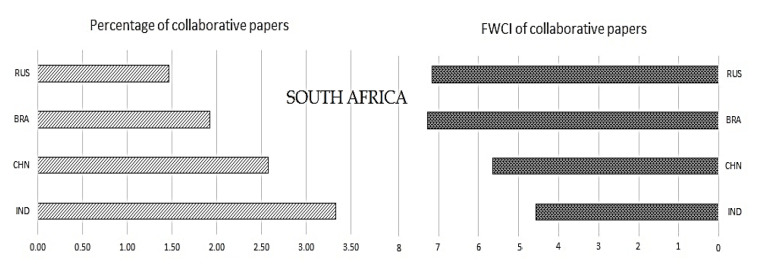
The interrelation of South Africa publication metrics with other BRICS countries.

Once we have understood the collaborative publications with the other regions, it is also necessary to understand the significance of collaboration mutually inside the BRICS nations. To understand the same, collaborative publications and their impact in terms of FWCI are plotted in Figures 9 to 13. Figure 9 shows the collaboration of Brazil with other BRICS nations and its impact. Similarly, Figure 10 shows the relation of Russia with other BRICS nations, followed by India in Figure 11, China in Figure 12, and South Africa in Figure 13.

**Brazil:** Brazil has around 0.98% of publications collaborating with India; these collaborative publications have a FWCI of 4.7. Around 0.76% of publications were co-authored with Chinese researchers, with a FWCI of 5.7. Coauthored publications with South Africa make up around 0.58%; these papers have a FWCI of 7.2. Collaborative publications with Russia make up around 0.42% of the total number of papers published by Brazil, with a FWCI of 7.1. It is observed that Brazil has a large number of collaborative works with India as compared to other BRICS nations and the highest impact is from collaborative work with South Africa.

**China:** China has around 0.85% of its total papers published with coauthors from BRICS nations. Russian collaborative work is at 0.27%, followed by India with 0.26%, Brazil with 0.18%, and South Africa with 0.11%. If FWCI is considered, publications with South Africa have a FWCI of 5.8, which is highest among the collaborations with BRICS nations. This is followed by collaborations with Brazil with a FWCI of 5.2 and Indian collaborative work with a FWCI of 4. Collaborative work with Russia has a FWCI of 3.7.

**India:** India produces around 0.92% of its publications in collaboration with China, which is the highest among all other BRICS nations, and its collaborative papers have a FWCI of 3.98. Around 0.5% of publications are South African collaborations, with a FWCI of 4.6. Of the total number of Indian publications, 0.43% are Brazil collaborations with a FWCI of 6.2, which is the highest value in comparison to collaborative publications with other BRICS nations. India has around 0.42% of its collaborative publications with Russia; these papers have a FWCI of 5.2.

**Russia:** Russia has around 4.13% of publications in collaboration with other BRICS nations. Russia has around 1.92% of its publications with China, and these papers have a FWCI of 3.7. The percentage of collaborations of Russia with India and Brazil is almost the same at around 0.97% and 0.96%, respectively. These papers have yielded a FWCI of 5.2 and 5.8, respectively. Russia has around 0.48% of its publications in collaboration with South Africa; these papers yield a FWCI of 7.2. It is seen that Russia has the highest collaboration with China and the papers with the highest FWCI are from South African collaborations, with a FWCI of 7.2.

**South Africa:** South Africa has around 9.4% of its publications in collaboration with the rest of the BRICS countries, with India's collaboration being the highest at 3.48%, with a FWCI of 4.7. ChinaSouth Africa collaborative publications account for 2.55%, with the papers having a FWCI of 5.8. There are around 1.95% of papers published by South Africa in collaboration with Brazil, with a FWCI of 7.3. Around 1.495% of the publications are with Russian collaborators; these papers have a FWCI of 7.1.

It can be understood that from the above graphs in Figure 9 to Figure 13 that South Africa shares a larger percentage of its publication with other BRICS nations, which is around 9.4% of its total publications. On the other hand, China has the lowest percentage of collaborative publications with other BRICS nations of 0.85%. Collaborative publication with South Africa has a higher FWCI as compared to other BRICS nations.

### Publication output in high impact journals

**Figure 14.**
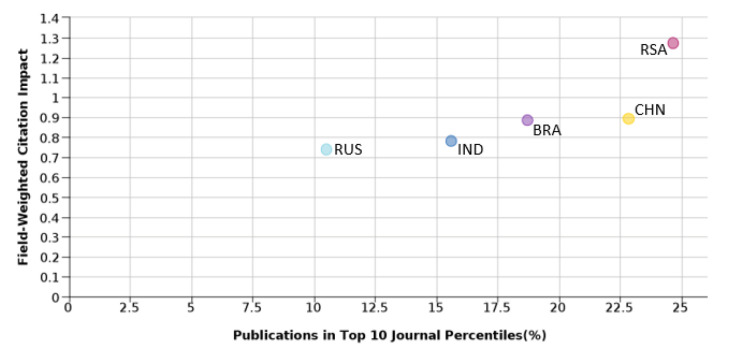
Comparison of quality of publication with publication-quality of BRICS countries. RSA: South Africa; BRA: Brazil; RUS: Russia; IND: India; CHN: China.

Along with collaboration, one more important parameter that also affects the quality of the publication is the choice of journal. For the analysis, the publication of BRICS nations in the journals that are in the top 10th percentile by citation score is compared with the quality in terms of FWCI. Figure 14 shows the outcome of BRICS nations in the top 10th percentile journals. It was found that 24.8% of South African publications are in the top 10th percentile journals with the highest FWCI of 1.39. Russia has the lowest percentage of publications in the top 10th percentile journals and its FWCI is also lowest at 0.73. India has around 15.5% of its publications in top 10th percentile journals with a FWCI of 0.79. Brazil, with a FWCI of 0.89, publishes 19.1% of its publications in the top 10th percentile journals. China produces around 22.8% of its publications in the top 10th percentile journals with a FWCI of 0.9. From the above discussions, it is clear that collaborative publication and journal quality are factors that would affect the outcome of the publication. Along with these two parameters, one more factor affecting the outcome is the subject area of the journals where papers are published.

### Subject area

**Figure 15.**
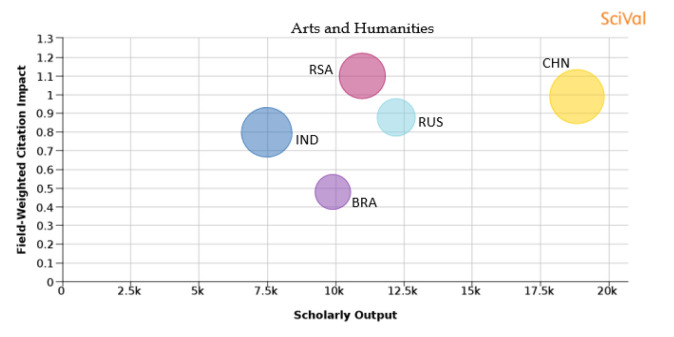
Comparison of quality of publication with publication quality and scholarly output in the subject area of Arts andHumanities. RSA: South Africa; BRA: Brazil; RUS: Russia; IND: India; CHN: China.

**Figure 16.**
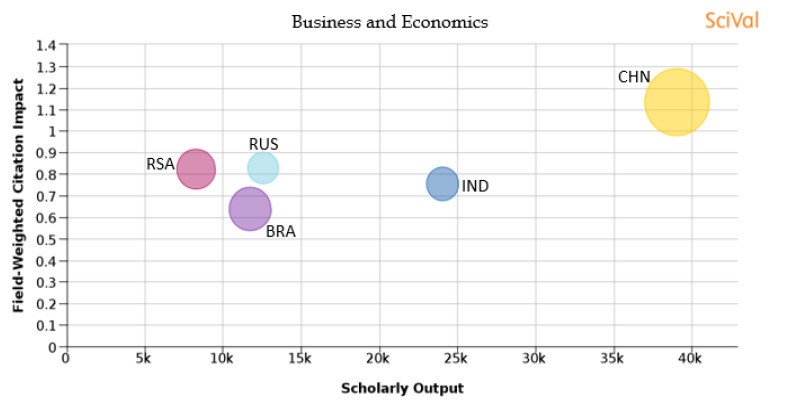
Comparison of quality of publication with publication quality and scholarly output in the subject area of Business and Economics. RSA: South Africa; BRA: Brazil; RUS: Russia; IND: India; CHN: China.

**Figure 17.**
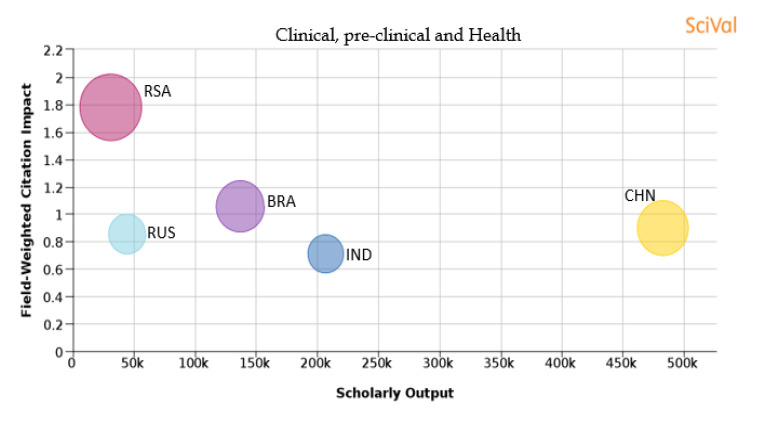
Comparison of quality of publication with publication quality and scholarly output in the subject area of Clinical, preclinical, and health. RSA: South Africa; BRA: Brazil; RUS: Russia; IND: India; CHN: China.

**Figure 18.**
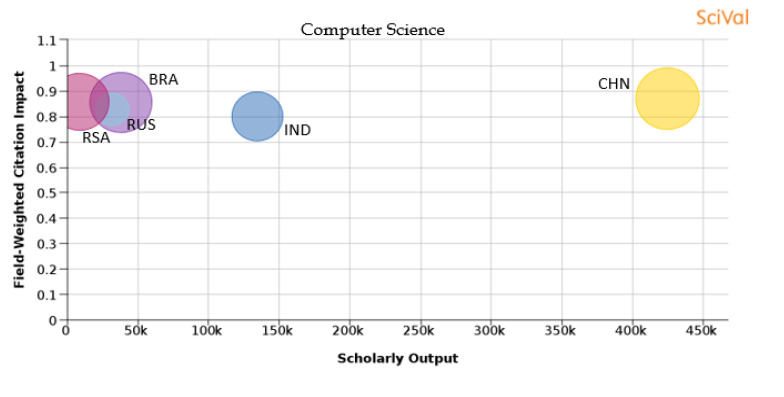
Comparison of quality of publication with publication quality and scholarly output in the subject area of Computer sciences. RSA: South Africa; BRA: Brazil; RUS: Russia; IND: India; CHN: China.

**Figure 19.**
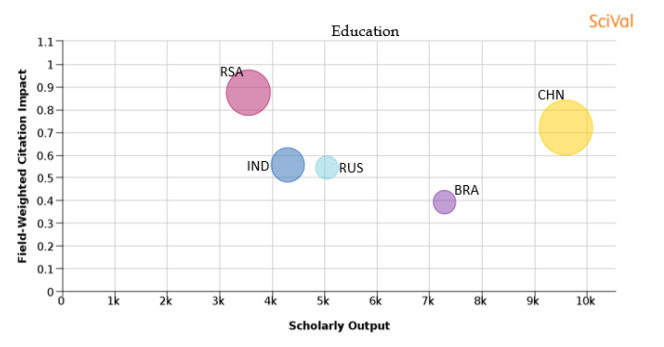
Comparison of quality of publication with publication quality and scholarly output in the subject area of Education. RSA: South Africa; BRA: Brazil; RUS: Russia; IND: India; CHN: China.

**Figure 20.**
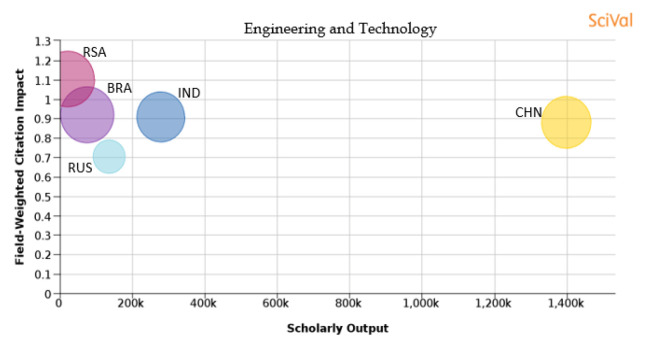
Comparison of quality of publication with publication quality and scholarly output in the subject area of Engineering and Technology. RSA: South Africa; BRA: Brazil; RUS: Russia; IND: India; CHN: China.

**Figure 21.**
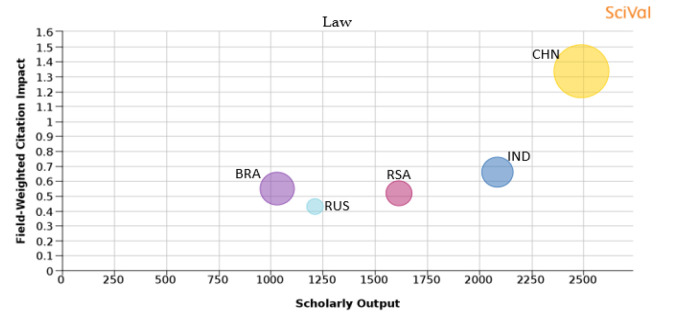
Comparison of quality of publication with publication quality and scholarly output in the subject area of Law. RSA: South Africa; BRA: Brazil; RUS: Russia; IND: India; CHN: China.

**Figure 22.**
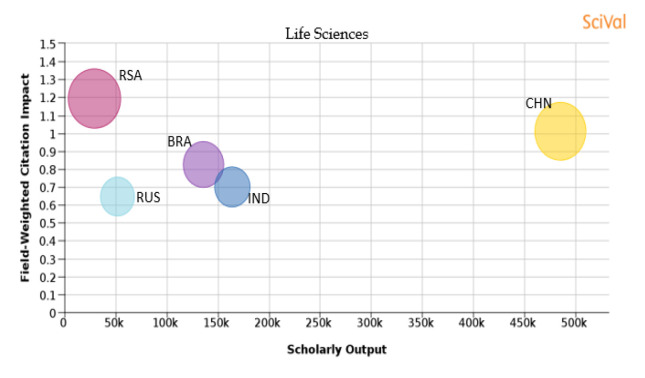
Comparison of quality of publication with publication quality and scholarly output in the subject area of Life Science. RSA: South Africa; BRA: Brazil; RUS: Russia; IND: India; CHN: China.

**Figure 23.**
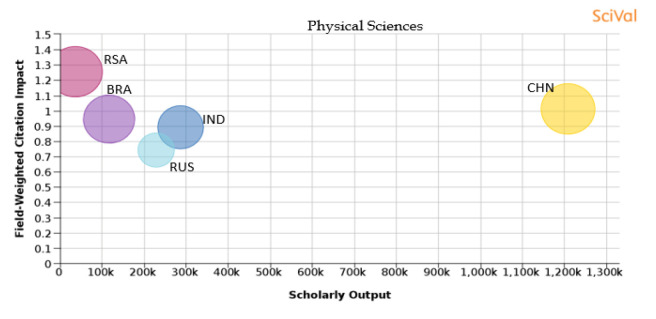
Comparison of quality of publication with publication quality and scholarly output in the subject area of Physical sciences. RSA: South Africa; BRA: Brazil; RUS: Russia; IND: India; CHN: China.

**Figure 24.**
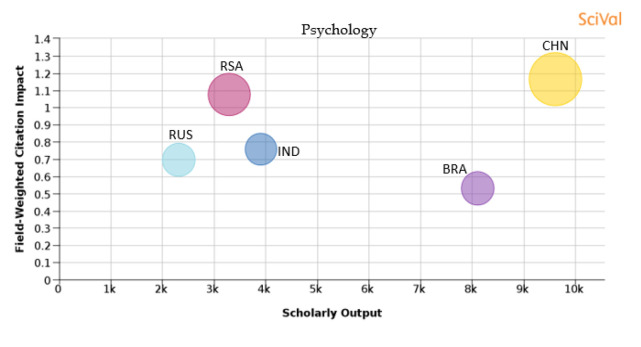
Comparison of quality of publication with publication quality and scholarly output in the subject area of Psychology. RSA: South Africa; BRA: Brazil; RUS: Russia; IND: India; CHN: China.

**Figure 25.**
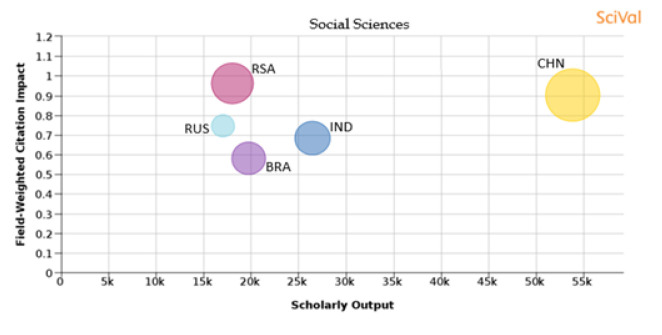
Comparison of quality of publication with publication quality and scholarly output in the subject area of Social science. RSA: South Africa; BRA: Brazil; RUS: Russia; IND: India; CHN: China.

The choice of journal for publishing a research article is becoming very important as it relates to the outcome of the publication in terms of research visibility and citation. The selection of journals based on subject area is also an important parameter that needs to be considered. As metrics like FWCI are normalized parameters across a given subject area, the outcome of the publication would depend on the subject area. Based on the present-day scenario, journals are categorized into 300+ sub-subject areas by Scopus. For the present analysis, we are considering the broad classification as followed by THE, where all journals are divided into 11 main subject topics, which are ‘arts and humanities’, ‘business and economics’, ‘clinical, pre-clinical and health’, ‘computer science’, ‘education’, ‘engineering and technology’, ‘law’, ‘life sciences’, ‘physical sciences’, ‘psychology’ and ‘social sciences’. In this classification, the available 300+ sub-subject area journals are mapped to these 11 areas. It is to be noted that the journals can be overlapping with one or more classified areas as many journals publish work on multi disciplinarily or interdisciplinarity research areas. Table 1 shows the percentage distribution of publications of each country over the 11 subject areas. An analysis is also shown in Figures 15 to 25 of each subject area and the BRICS countries’ publication outputs, publication quantity, and quality of the publications.

**Table 1.** Break up of publication output across subject areas.

Name	Brazil (%)	China (%)	India (%)	Russia (%)	South Africa (%)
Arts and Humanities	2.38	0.66	0.94	3.14	9.09
Business and Economics	2.81	1.37	3.01	3.23	6.84
Clinical, pre-clinical and health	32.83	16.91	25.89	11.35	25.71
Computer Science	9.10	14.89	16.88	8.21	7.29
Education	1.75	0.34	0.54	1.30	2.93
Engineering and Technology	18.15	48.94	34.93	34.70	17.28
Law	0.25	0.09	0.26	0.31	1.34
Life Sciences	32.68	16.99	20.54	13.22	24.08
Physical Sciences	27.98	42.37	35.92	58.79	29.76
Psychology	1.95	0.34	0.49	0.60	2.72
Social Sciences	4.75	1.89	3.31	4.38	14.93
Arts and Humanities	2.38	0.66	0.94	3.14	9.09
Business and Economics	2.81	1.37	3.01	3.23	6.84

From the table, it is seen that the split of publications across all the 11 subject areas is not equal and also varies by country. Brazil as a country produces a large share of publications in the subject areas of ‘clinical, pre-clinical and health’, ‘life science’ and ‘physical science’, and the least number of publications is seen in the subject area of ‘law’. In the case of China, a large share of publications is in the subject areas of ‘physical science’ and ‘engineering and technology’, and the least number of publications is from the subject areas of ‘law’, ‘education’, ‘psychology’, ‘arts and humanities’. India has a large share of publications in ‘physical sciences’, ‘engineering and technology’, and ‘clinical, pre-clinical and health’ subjects and lowest share of publications in the areas of ‘law’, ‘psychology’, ‘education’, and ‘arts and humanities’. Russia has a large share of publications in ‘Physical Sciences’, ‘Engineering and Technology’, with the lowest share of publications in the area of ‘psychology’ and ‘law’. South Africa has a large share of publications from ‘physical sciences’, ‘clinical, pre-clinical and health’, ‘life sciences’ and least share of publication in ‘law’ ‘psychology’ and ‘education’.

**Arts and humanities:** Figure 15 shows the output of BRICS nations in the subject area of ‘arts and humanities’ for the period 2012-17. China produces a large number of papers in ‘arts and humanities’, whereas the percentage share of South African papers is the highest. The FWCI of South African publications is the highest with a value of 1.1, followed by China with 0.99, Russia with 0.88, India with 0.8, and Brazil with 0.48. Publication of papers in the top 10th percentile journals is highest in China with 22%, followed by India with 17.6%, China with 15.5%, Russia with 10.2%, and Brazil 8.8%. It can be seen that China, with a higher percentage of papers in the top 10th percentile journals, also has a higher FWCI, and Brazil with less publications in the top 10th percentile journals has a lower FWCI.

**Business and economics:** China produces a large number of papers in the subject area of ‘business and economics’. South Africa has a large share of its publications in ‘business and economics’. China also produces 28.4% of its publications in the top 10th percentile journals and has the highest FWCI of 1.13. Brazil has around 12% of its publications in the top 10th percentile journals with a FWCI of 0.64. South Africa produces around 10.2% of its publications in the top 10th percentile journals with a FWCI of 0.82. The FWCI of Russian publications is 0.83 with around 6.2% in the top 10th percentile journals. Around 7% of publications from India are in the top 10th percentile journals with a FWCI of 0.75, as evident in Figure 16.

**Clinical, pre-clinical, and health:** China produces the highest number of papers in the subject area of ‘clinical, pre-clinical and health’ which is around 470,000, but Brazilian publications have a higher percentage share in this subject area as compared to other countries as shown in Table 1. South African publications have the highest FWCI quality parameter of 1.78 with the highest percentage of publications in the top 10th percentile journals (25.2%). Brazilian publications have the next highest FWCI of 1.06, with 15.5% of its publications in the top 10th percentile journals. The FWCI of Chinese publications is 0.9, with 17.3% of publications in the top 10th percentiles journals. Publications from Russia have a FWCI of 0.86, with 8.9% of its publications in the top 10th percentile journals. India has a lower FWCI of 0.71 in comparison to other BRICS nations, with 8.6% of its publications in journals that are in the top 10th percentile. It is observed that countries with more publications in the top 10th percentile journals have a higher FWCI as shown in Figure 17.

**Computer science**: It can be observed from Figure 18 that in the subject area of ‘computer science’, the publication number of China is the highest. India and China have a higher share of publications as compared to other BRICS nations. The behavior observed is unique and different as compared to other subject areas where we see that almost all nations have similar values of FWCI ranging 0.87 to 0.83. The percentage of publications in top 10th percentile journals is 11.5% for China, followed by Brazil with 11.2%, South Africa with 9.8%, India with 7.4%, and Russia with 3.3%.

**Education:** Publications from South Africa have a FWCI of 0.88, which is the highest of all the BRICS nations, and 9.4% of publications of South Africa are in the top 10th percentile journals. China has around 13.4% of its publications in the top 10th percentile journals, with a FWCI of 0.72. India, with 5.45% of its publications in the top 10th percentile journals, has a FWCI of 0.56. Russia, with 2.5% of its publications in the top 10th percentile journals, has a FWCI of 0.54, and Brazil also has around 2.55% of its publications in journals of the top 10th percentile as indicated in Figure 19.

**Engineering and technology:** From Figure 20, it can be seen that the number of publications from China is the highest in the subject area of ‘engineering and technology’ in comparison to other BRICS nations. Publications from South Africa have the highest FWCI among other BRICS nations at 1.1, and their percentage of publications in the top 10th percentile journals is also highest at 24%. Brazil has around 23.8% of its publications in the top 10th percentile journals with a FWCI of 0.92, followed by India whose publications have a FWCI of 0.91 and has 19.6% of its publications in top 10th percentile journals. China has around 20.1% of its papers in the top 10th percentile journals, and the publications from China have a FWCI of 0.88. Russia, at 8.8%, has the lowest percentage of publications in the top 10th percentile journals and the lowest FWCI at 0.71.

**Law:** Law as a subject has a very small share of publications in all the BRICS nations as compared to the other subject areas, with South Africa having the highest share of 1.34% and China having the lowest share at 0.09%, although in terms of absolute number, the number of publications from China is higher in comparison to other BRICS nations. The quality of publications in terms of FWCI is also higher in papers produced by China (1.3), and it is also seen that around 50% of papers published are in journals that are in the top 10th percentile. The percentage of publications from India in the top 10th percentile journals is about 16.6% and the FWCI of papers published from India is 0.66. Papers published from Brazil have a FWCI of 0.55 and 19% of its publications are in the top 10th percentile journals. South Africa has around 16.6% of its publications in the top 10th percentile journals and the FWCI of South African publications is 0.52 for ‘law’. Russia has the lowest percentage of papers published in the top 10th percentile journals, which is 4.5%, and the FWCI of Russian papers in law has the lowest FWCI of 0.43 as compared to other BRICS nations as shown in Figure 21.

**Life science:** From Table 1 it is understood that Brazil has a large share of publications in the subject area of life science, while China has a higher number of publications. South Africa has around 26.4% of its publications in top 10th percentile journals and the FWCI of the papers published is 1.2, which is highest in all BRICS countries. China has around 24.6% of its publications in the top 10th percentile of journals and the published papers have a FWCI of 1.01. Around 16.3% of the publications from Brazil are in journals which are in top 10th percentile, and published papers have a FWCI of 0.82. The FWCI of papers published from India is 0.7 with 11.7% of papers in the top 10th percentile journals. Of papers published from Russia, 11.3% are published in journals which are in the top 10th percentile, and papers published have a FWCI of 0.65, which is lowest among all the BRICS nations. It can be seen that in all these nations the quality of the publication is proportional to the number of publications in the top 10th percentile journals as displayed in Figure 22.

**Physical science:** In the area of physical science, as shown in Figure 23, China has the highest number of publications, while the share of publications in Russia is the highest. Publications from South Africa have the highest percentage of publication in the top 10th percentile journals (22.8%), with publication having a FWCI of 1.26. Around 22.3% of publications are in the top 10th percentile journals in China and publications have a FWCI of 1.01. Around 20.3% of papers Brazil produces are published in the top 10th percentile journals and papers have a FWCI of 0.95. The FWCI of papers published by India is 0.89, and they publish around 16.2% of their papers in top 10th percentile journals. Papers from Russia have a FWCI of 0.74 with 10.2% of its publications in the top 10th percentile journals.

**Psychology:** Publications from China have around 26.7% of papers in the top 10th percentile journals and the FWCI of papers published is 1.17. In this subject area, 16.8% of South African papers were published in the top 10th percentile journals, and the FWCI of the papers published was 1.08. Publications from India have a FWCI of 0.76, with 9.8% of papers published in the top 10th percentile journals. Brazil published around 10.7% of its publications in the top 10th percentile journals, and publications from Brazil had a FWCI of 0.53, which is the lowest compared to other BRICS nations. Russia produced around 10.5% of its publications in the top 10th percentile journals and the FWCI of the papers published by Russia was 0.7, as shown in Figure 24.

**Social sciences:** From Figure 25, it is seen that South Africa publications have a FWCI of 0.96, with 16.5% of its publications in the top 10th percentile journals. In China around 26.6% of papers have been published in the top 10th percentile journals, and papers published have a FWCI of 0.9. Russia has 4.8% of its papers published in the top 10th percentile journals and papers have a FWCI of 0.75. The FWCI of papers published by India is 0.68 and around 11.3% of its publications are in the top 10th percentile journals. Brazil has the lowest FWCI at 0.58 and around 10.2% of their publications are in the top 10th percentile journals.

The publication numbers of China is the highest compared to other BRICS nations in all the 11 subject areas. The FWCI of publications from South Africa is highest in the subject areas of ‘arts and humanities’, ‘clinical, pre-clinical and health’, ‘education’, ‘engineering and technology’, ‘life sciences’, ‘physical sciences’ and ‘social sciences’. For the subject areas ‘business and economics’, ‘computer science’, ‘law’ and ‘psychology’, publications from China have the highest FWCI. From the above discussion, it is clear that the quality of publication depends on multiple parameters like collaboration, publication in quality journals, and subject area of publication.

## Discussion

Research and innovation are the parameters that often decide the growth of an institute or organization or country. Publication output is one of the measures by which a country’s research can be quantified. An attempt was made in this paper to study the behavior of publications of the developing countries Brazil, Russia, India, China, and South Africa (BRICS). To validate the analysis of the work reported, the number and position of universities ranked in THE were treated as a unit of measure. It is evident from the publication that China which has the higher number of publications, research spend, researcher population, has a greater number of universities ranked. On the other hand, SouthAfrica with lesser publication outcome, research spend, etc. has lower number of universities ranked. But it can also be seen that the percentage of universities ranked higher due to higher quality parameters like higher FWCI, international collaboration, etc. Though the number of universities being ranked is higher due to higher researcher volume and publication the quality parameters reflect on the percentage of universities ranked higher. To understand the behavior of publication output, only Scopus-indexed sources were considered as THE considers only Scopus-indexed publications. The first parameter to be evaluated was the population of researchers and it was found that China has the largest population also produces the highest number of publications; the relationship between researcher population and quantity of publications was evident. The second parameter to be analyzed was the percentage of GDP spent on research and though it was expected that it would be proportional to the quality and number of publications, in reality, it was not so, leading to further analysis to find the other parameters contributing to the outcome. One such measure was collaborative publications, and from this analysis it was evident that South Africa with the most collaborative research had a higher impact than the rest of the BRICS nations. The pattern of collaboration was also studied to understand the geography of collaboration. Results show that South Africa, Russia, and Brazil have more collaboration with Europe, China with North America, and India showed broader collaboration.

The focus area of each of the countries is unique to its culture, geography, and relevance to society. An attempt was made to understand the impact of focus area in terms of the 11 basic subjects on the quantity and quality of research. The nations showed an inclination to a few research areas; for example, Brazil had a large focus toward clinical, preclinical, and life sciences. China and India had a large focus on engineering and physical sciences. Russia showed large publication numbers (more than 50% of its publications) in the areas of physical sciences, followed by engineering with 34%. South Africa showed equal priorities to clinical, life science, and physical science. It was also found that the fewest publications were from law. The paper tried to report the behavioral analysis of research in the BRICS nations.

### Data availability

All data underlying the results are available as part of the article and no additional source data are required.
