# Supplementary material for: A bibliometric study on the research outcome of Brazil, Russia, India, China, and South Africa
Source: F1000Res. 2021 Mar 16;10:213. [Version 1] doi: 10.12688/f1000research.51337.1 (PMC8174118; doi:10.12688/f1000research.51337.1)
Supplement: Peer review history

# Peer review: Reviewer response for version 1

**DOI:** 10.5256/f1000research.54493.r85737

**Published:** 2021-06-02

**Authors:** Vipan K. Parihar

**Affiliations:** 1 Department of Radiation Oncology, University of California, Irvine, Irvine, CA, USA; 2 NASA Specialized Center for Research, University of California, Irvine, Irvine, CA, USA

I found this article interesting and insightful and particularly well written. The authors have done a noteworthy study to gauge the scientific input and its impact on development in BRICS countries. As such, for this manuscript, the authors have used multiple sources of data namely World Bank, Scopus, and also from Elsevier which is praiseworthy and is quite elaborate. This kind of bibliometric study is needed to bring the focus on core areas of development that will help in 21st-century nation-building. It also helps to show gaps, and improve research thrust and intensify research pursuits in those areas which lack focus which need some special stimulus to kick start research. These will help governments and policymakers to take note of the direction in which future research should be shaped. 

 In my opinion, if the authors would have correlated this data with some more relevant info on commercialization efforts (patents), the number of millionaires, HNWI, thrown in with data on AI, 5G/6G internet connectivity for good measure it would have been a comprehensive study although quite complicated which would need more time and detailed statistical analysis to pool the results and come to a meaningful conclusion.

 Anyway, I congratulate the authors for choosing this study impacting the BRICS.

Is the work clearly and accurately presented and does it cite the current literature?

Yes

If applicable, is the statistical analysis and its interpretation appropriate?

Yes

Are all the source data underlying the results available to ensure full reproducibility?

Yes

Is the study design appropriate and is the work technically sound?

Yes

Are the conclusions drawn adequately supported by the results?

Yes

Are sufficient details of methods and analysis provided to allow replication by others?

Yes

Reviewer Expertise:

My research is motivated by the need to better understand the pathophysiology of brain dysfunction. Specifically, my interests include evaluating the sex differences in brain structure, chemistry, and function. The long-term goal of my research is to develop clinically applicable strategies that are efficient for enhancing brain function after injury, disease, or aging.

I confirm that I have read this submission and believe that I have an appropriate level of expertise to confirm that it is of an acceptable scientific standard.

# Peer review: Reviewer response for version 1

**DOI:** 10.5256/f1000research.54493.r81649

**Published:** 2021-04-21

**Authors:** Alka Ahuja

**Affiliations:** 1 College of Pharmacy, National University of Science and Technology, Muscat, Oman

The article highlights comparison of BRICKS countries in terms of quality publications in various fields of Research. A very interesting study done in a meticulous and unbiased way based on THE data, which is globally accepted. More citations from 2020 could have been added. The figures summarize the findings very well. The comparison is based on development expenditure, publication quality, international collaborations etc. I recommend indexing of this article.

Is the work clearly and accurately presented and does it cite the current literature?

Yes

If applicable, is the statistical analysis and its interpretation appropriate?

Yes

Are all the source data underlying the results available to ensure full reproducibility?

Yes

Is the study design appropriate and is the work technically sound?

Yes

Are the conclusions drawn adequately supported by the results?

Yes

Are sufficient details of methods and analysis provided to allow replication by others?

Yes

Reviewer Expertise:

Pharmaceutics , Health outcomes, Pharmaceutical Management, Publication Ethics

I confirm that I have read this submission and believe that I have an appropriate level of expertise to confirm that it is of an acceptable scientific standard.
